# Amyloid Beta Oligomers as Early Triggers of Neuronal Cytoskeleton Dysfunction in Alzheimer’s Disease

**DOI:** 10.3390/pathophysiology33010014

**Published:** 2026-02-03

**Authors:** Yadira Gasca-Martínez, Miguel Angel Ontiveros-Torres, Isaías López-Gallegos, José Jaime Jarero-Basulto

**Affiliations:** 1 Departamento de Biología Celular y Molecular, Universidad de Guadalajara, Zapopan 45200, Jalisco, Mexico; yadira.gasca3953@alumnos.udg.mx (Y.G.-M.); isaias.l.g.w@gmail.com (I.L.-G.); 2 Escuela de Ingeniería y Ciencias, Tecnológico de Monterrey, Monterrey 64849, Nuevo León, Mexico; miguelontiveros@tec.mx

**Keywords:** Alzheimer’s disease, amyloid beta, oligomers, aggregates, cytoskeleton

## Abstract

Alzheimer’s disease (AD) is characterized by progressive cognitive decline, with amyloid beta oligomers (AβOs) emerging as the most neurotoxic species and acting as early triggers of cellular alterations. Before the appearance of other protein aggregates, AβOs disrupt the dynamics and stability of the neuronal cytoskeleton, a structure essential for maintaining neuronal morphology, axonal transport, and synaptic plasticity. Experimental evidence demonstrates that AβOs promote microtubule disassembly, Tau hyperphosphorylation, reduced kinesin levels, impaired axonal transport, and alterations in actin dynamics through the LIMK–cofilin signaling pathway. In addition, increased levels of neurofilament light chain have been identified as an early biomarker of axonal damage. Notably, these cytoskeletal disturbances arise in the absence of extensive neuronal death, underscoring the cytoskeleton as a critical early target in AD pathogenesis. In this review, we analyze cytoskeletal alterations induced by AβOs in neurons and discuss how these changes may contribute to disrupted neuronal communication, a defining early hallmark of AD pathology.

## 1. Introduction

Alzheimer’s disease (AD) is a chronic and progressive neurodegenerative disorder that accounts for approximately 60–80% of dementia cases in the elderly population. Current global estimates indicate that more than 50 million individuals are affected by AD, and this number is expected to increase substantially in the coming decades, with a disproportionate impact on developing countries. Clinically, AD manifests as a gradual decline in cognitive abilities and overall mental capacity [1,2]. Although the precise etiology of AD remains unclear, the prevailing hypothesis suggests that the accumulation of aberrant Tau and amyloid beta (Aβ) proteins in the brain drives disease onset and progression [3,4]. Nevertheless, the causal relationship between abnormal protein aggregation and cognitive impairment has not been fully elucidated.

Over the last decades, oligomeric aggregates of both Tau and Aβ have gained greater pathological relevance than their fibrillar forms, as they are now regarded as the major neurotoxic species involved in the initiation and progression of AD [5,6]. In particular, increasing evidence indicates that Aβ oligomers (AβOs) play a critical role in the early stages of the disease, when cellular and morphological alterations emerge in the absence of other types of protein aggregates and before of intense neuronal death [7,8]. In this context, elucidating the relationship between AβO formation, cytoskeletal disorganization, and early AD progression becomes essential, since the cytoskeleton is one of the most vulnerable cellular components in the presence of these toxic aggregates [9,10]. However, investigating the pathological relationship among AβOs, the cytoskeleton, and AD in human brain tissue remains challenging, because AβOs formation begins many years before symptom onset, when neuronal loss is still minimal and therefore difficult to detect (presymptomatic stage). Nonetheless, several reports indicate that during early stages of AD, impairments in intercellular communication and intracellular transport occur well before widespread neuronal loss. These subcellular disturbances are directly associated with cytoskeletal alterations, suggesting that cytoskeletal dysfunction represents a pivotal pathological event in AD progression [11].

To date, most neuropathological studies in human have focused on advanced stages of AD, in which extensive tissue damage obscures early pathogenic events. Although analyses of preclinical patients could provide a promising approach to investigate these initial alterations, identifying such cases remains highly challenging [12]. On the other hand, most experimental models in animals or cultured cells have focused on evaluating the cytotoxicity of protein aggregates, mechanisms of cell death, or potential therapeutic compounds. However, only few studies have specifically addressed the early alterations triggered by AβOs formation and their interaction with cytoskeletal components.

In this review, we examine experimental evidence supporting cytoskeletal modifications induced by AβOs as key events in AD pathogenesis. We first summarize current knowledge regarding the amyloid protein precursor (APP), the Aβ peptide, and the formation of AβOs, and then discuss the structural and functional cytoskeletal alterations associated with synaptic dysfunction. Finally, we emphasize the importance of exploring and understanding these early events and their consequences prior to neuronal loss, as this knowledge may contribute to the development of strategies aimed at preserving or restoring cytoskeletal organization and dynamics.

## 2. Search Method and Criteria for Literature Inclusion

A search of the MEDLINE database via PubMed (http://pubmed.ncbi.nlm.nih.gov) was conducted to identify articles published up to August 2025 addressing AβOs (specifically Aβ1-42) and their early effects on cytoskeleton components in the context of AD. The search strategy included the following keywords and phrases: “Aβ oligomers and cytoskeleton”, “cytoskeletal alterations induced by Aβ oligomers”, “Alzheimer’s disease and cytoskeletal alterations” and “AD”, as well as combination of these terms. Only research articles reporting the effects of AβOs on cytoskeletal organization in neural cells were included. Eligible studies comprised both in vitro experiments and in vivo models of AD. No restrictions were applied regarding species or age in the case in in vivo studies, given the narrative nature of this review.

## 3. Amyloid Precursor Protein and Amyloid Beta Peptide

Although the origin of AD remains unknown, accumulating experimental evidence supports the notion that abnormal protein aggregation represents a critical factor in its development. In neurodegenerative disorders, including AD, the challenge is not only to identify the protein that initiates the pathological cascade, but also to delineate the specific aggregate species that mediate cellular toxicity. A key consideration is that pathogenic proteins can adopt multiple aggregation states depending on the number of assembled molecules, giving rise to species that differ in size, morphology, stability, conformation, and toxicity.

In this context, Aβ is a protein of considerable pathological interest, as several of its aggregation forms are associated with the onset, or at least the early stages of AD. Observations from human patients and experimental models led to the formulation of the amyloid cascade hypothesis [13], which although challenged for its inability to fully explain several unresolved aspects of the disease, remains a fundamental conceptual framework for understanding AD pathogenesis [14].

The APP of Aβ, is a type I transmembrane protein localized in the plasma membrane and other membranous organelles. It is widely expressed in many tissues, although its expression is particularly high in the brain [15]. Although APP is considered a key element in AD pathophysiology, it also plays important roles in neuronal cell physiology, including cell cycle regulation, neurogenesis, synaptic mechanisms, cell differentiation, among other processes [16,17]. The biological importance of APP is supported by studies in animal models in which APP deletion or modification results in a wide range of brain alterations [18,19].

In humans, the APP gene is localized on chromosome 21 [20] and, through alternative splicing generates between 8 and 11 isoforms depending on the tissue, with variable lengths [21]. Among these, APP695, APP751 and APP770 are the predominant isoforms. APP751 and APP770 are mainly expressed in glial cells, whereas APP695 is predominantly found in neurons [22]. Although all isoforms are capable of producing Aβ, APP751 and APP770 contain a Kunitz-type protease inhibitor (KPI) domain, which is associated with the regulation of APP proteolytic processing and increased Aβ production [23].

In general, APP is composed of three domains: a large extracellular region, a single transmembrane segment and a short cytoplasmic tail. APP processing has been extensively characterized and involves a multistep mechanism governed by two major pathways: the amyloidogenic and the non-amyloidogenic routes. Both depend on the coordinated activity of three secretases. In the non-amyloidogenic pathway, APP is first cleaved by α-secretase, a zinc-dependent metalloproteinase. This cleavage generates two fragments: a soluble N-terminal extracellular domain (sAPPα) and a membrane-bound C83 fragment. Importantly, this proteolytic event precludes the formation of Aβ peptides because it occurs within the central region of the Aβ sequence, thereby preventing its subsequent generation. The sAPPα fragment, which is rapidly eliminated, has been shown to participate in several physiological processes, including early central nervous system development, neuroplasticity and neurogenesis [24,25]. Additionally, sAPPα can prevent Aβ formation by inhibiting β-secretase activity [15]. The C83 fragment is subsequently processed by γ-secretase, a membrane protein complex composed of presenilin 1 and 2, presenilin enhancer 2, nicastrin and anterior pharynx-defective-1. This cleavage yields two new fragments: the amyloid precursor protein intracellular domain (AICD) and the p3 peptide. AICD has been suggested to play a key role in gene signaling and transcriptional regulation, including APP processing itself [26,27]. Although the role of p3 in the extracellular space remains unclear, it has been associated with neuroprotective functions [28].

**Figure 1:**
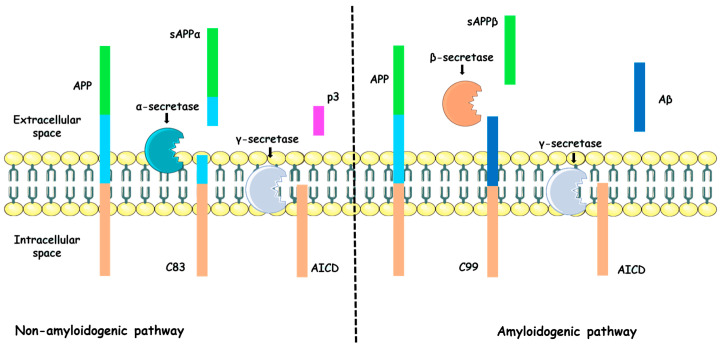
Schematic representation of APP processing pathways. The non-amyloidogenic pathway involves cleavage of APP by α-secretase followed by γ-secretase, preventing Aβ formation. In contrast, in the amyloidogenic pathway, β-secretase cleaves APP to generate sAPPβ, followed by a second cleavage by γ-secretase, resulting in the release of Aβ peptide into extracellular space (pathological pathway). Elements were adapted from Server Medical Art (https://smart.servier.com), licensed under Creative Commons Attribution 4.0 (https://creativecommons.org/licenses/by/4.0).

In contrast, in the amyloidogenic pathway, secretase homeostasis becomes deregulated in adulthood [29], leading to APP cleavage by β-secretase instead of α-secretase. β-secretase (also known as BACE-1), which is located in the extracellular space as well as in the membranes of the trans-Golgi network and endosomes, generates an N-terminal extracellular fragment (sAPPβ) and a second membrane-bound fragment (C99). Interestingly, experimental evidence has demonstrated that β-secretase silencing in animal models of AD results in the absence of Aβ peptide production [30,31]. Moreover, Sennvik and coworkers reported a high presence of sAPPβ surrounding neuritic plaques and brain blood vessels in AD patients [32]. The C99 fragment is subsequently proteolyzed by γ-secretase, producing AICD and releasing the Aβ peptide into the extracellular space (Figure 1) [15,22].

Interestingly, γ-secretase is not only present in the plasma membrane but is also found in mitochondrial, endoplasmic reticulum, lysosomal, and endosomal membranes [33,34]. This broad distribution supports the idea that APP processing occurs in multiple cellular compartments, increasing the endogenous presence of Aβ and, consequently, its pathological effects [35,36]. In this context, it has been proposed that the non-amyloidogenic pathway is primarily associated with the plasma membrane [37], whereas the amyloidogenic pathway predominantly occurs within the endosomal system [38], where cytoskeletal function is highly relevant. Although APP commonly follows the non-amyloidogenic route, under certain yet unknown circumstances it can be redirected toward the amyloidogenic pathway, favoring Aβ peptide generation and thereby increasing the probability of developing AD.

Aβ is a short peptide of approximately 37–43 amino acids in length [15]. Among its isoforms, Aβ1-42 is considered the most relevant from a pathological perspective due to its biochemical properties [39,40]. Experimental evidence has shown that the two additional amino acids in Aβ1-42, compared with Aβ1-40, facilitate its aggregation, which has been associated with increased toxicity [41]. Nevertheless, the main pathological issue is not Aβ formation per se, but rather its overproduction and deficient clearance, which contribute to its progressive accumulation and trigger a pathological cascade that involves Tau hyperphosphorylation, synaptic damage, neurodegeneration and cognitive decline [42,43].

## 4. Formation and Characteristics of AβOs

**Figure 2:**
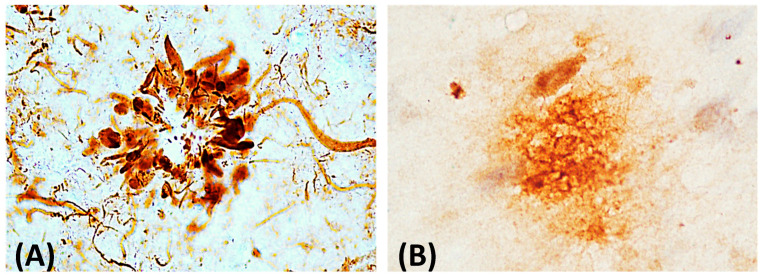
Representative Aβ deposits in AD. Bright-field immunohistochemical images of hippocampal tissue from AD patients. (**A**) Neuritic plaque composed of a dense Aβ core, surrounded by degenerating cellular processes. (**B**) Amyloid plaque characterized by extracellular Aβ accumulation with a typical circular distribution. Both structures were detected using specific antibodies at 40x magnification. The images are original to the authors’ image bank and illustrate advanced pathological Aβ lesions in the AD.

A major biochemical characteristic of the Aβ peptide is its intrinsic tendency to self-aggregate and form toxic structures of increasing structural complexity. These aggregates become progressively more stable and less susceptible to degradation, enabling them to exert damage on neuronal cells through multiple mechanisms. Therefore, understanding the process of Aβ aggregate formation is essential for elucidating the molecular basis of neurodegeneration in AD. Aβ can self-aggregate into several structural species, progressing from monomers to AβOs, protofibrils, insoluble fibrils and eventually amyloid plaques (Figure 2) [15,44], all of which have been detected through the combined use of different analytical techniques [23,45]. However, not all Aβ monomers follow the same aggregation route or necessarily generate identical structural products, complicating the prediction of their fate [46]. Clinical observations, together with multiple experimental models (including animal, cellular and biophysical) [15,47,48], have led different research groups to propose integrative models to explain the mechanism underlying Aβ aggregation, as described below.

**Figure 3:**
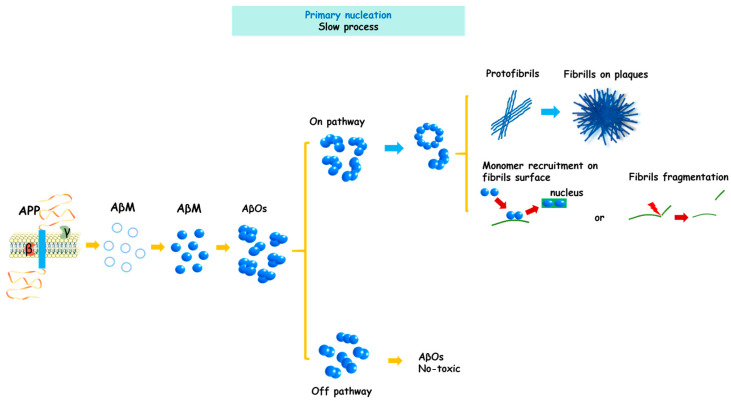
General model of Aβ peptide aggregation during AD. Following APP proteolysis, Aβ monomers are released and initially adopt an unstable α-helix conformation, which transitions into β-sheet-rich structures, favoring the formation of early soluble aggregates (AβOs). During primary nucleation, these AβOs act as seeds that initiate elongation into protofibrils and fibrils, a process referred to as the on-pathway. Mature fibrils, the main component of amyloid plaques, can fragment and generate new nuclei or act as transient surfaces that recruit monomers, which accumulate, undergo conformational changes, and are released as new nuclei. Both mechanisms accelerate toxic species production through secondary nucleation. In parallel, some AβOs cease elongation and remain in soluble, non-fibrillar forms, considered off-pathway species.

Following APP cleavage, Aβ released into the extracellular space initially adopts an unstable α-helix conformation that subsequently undergoes a transition into a β-sheet structure, in which three key regions of the peptide sequence are involved [49]. This β-sheet conformation facilitates hydrogen bond formation between Aβ peptides and promotes the formation of small soluble AβOs, which act as aggregation nuclei. Experimental evidence indicates that the C-terminal region of Aβ (residues 29–42) is highly hydrophobic, favoring its insertion into lipid bilayers, particularly within lipid rafts enriched in sphingomyelin, cholesterol and GM1 ganglioside. Within these membrane microdomains, Aβ peptides become concentrated and stabilized, facilitating the formation of small AβOs that can subsequently be released and act as new aggregation nuclei [50]. These events, collectively referred to as primary nucleation, represent the first critical step in the fibrillogenic pathway [22,51]. As AβOs incorporate additional Aβ monomers, protofibrils emerge as characteristic “bead-like” structures [52], followed by the formation of insoluble fibrils [53,54], which ultimately assemble into amyloid plaques (Figure 3). AβOs that follow this progression are classified as “on-pathway” species. In contrast, soluble AβOs that do not advance toward insoluble fibril formation are considered “off-pathway” species and have been presumed to be less toxic [47], although their study remains limited due to difficulties in structural characterization, even using high-resolution techniques such as atomic force microscopy (AFM) or electron microscopy [55].

During on-pathway aggregation, Aβ kinetics are typically described as occurring in two phases: a slow nucleation phase followed by a fast elongation phase [56,57]. Elongation generates protofibrils that according to Yang and coworkers [58], adopt an S-shape conformation critical for their transition into fibrils. These fibrils elongate through the incorporation of Aβ monomers at their ends, forming the characteristic cross-β sheet arrangement essential for structural stability [59,60]. For many years, Aβ fibrils were considered the main toxic species due to their stability and resistance to degradation. However, attention has shifted toward intermediate oligomeric species, which are now recognized as significantly more harmful. Although fibrils are not excluded as pathological elements, their role is currently interpreted as platforms that facilitate the formation of new AβOs or nuclei. In this regard, fibrils have been shown to fragment and release soluble AβOs, creating new elongation sites and accelerating the aggregation [61,62]. Additionally, fibrils can catalyze the formation of new AβOs by retaining and converting monomers, which are subsequently released as new nuclei. Both processes drive the production of toxic species through a mechanism known as secondary nucleation [63]. These findings indicate that the greatest production and structural diversity of AβOs arises during secondary nucleation [64]. Although primary nucleation generates fewer AβOs, these can still contribute to early cellular alterations.

AβOs can adopt multiple conformations, including globular, annular or β-barrel-like structures, enabling them to interact with specific receptors or insert into cellular membranes to form conductive pores, thereby disrupting cellular physiology [65]. In addition, AβOs isolated from human AD brains have been shown to act as seeds capable of inducing conformational changes in Aβ in vitro. In parallel, studies in cellular and animal models have demonstrated that AβOs can propagate through prion-like mechanism [66,67], a behavior that may explain their spread across neuronal networks [55,68,69]. Consequently, multiple studies conclude that prefibrillar AβOs, rather than insoluble fibrils, represent the truly pathogenic species in AD [70,71]. Altogether, these findings underscore the importance of elucidating the pathological mechanisms governing the formation and propagation of AβOs, which may support the development of novel therapeutic strategies for AD and other neurodegenerative disorders.

## 5. Structure and Function of the Neuronal Cytoskeleton

**Figure 4:**
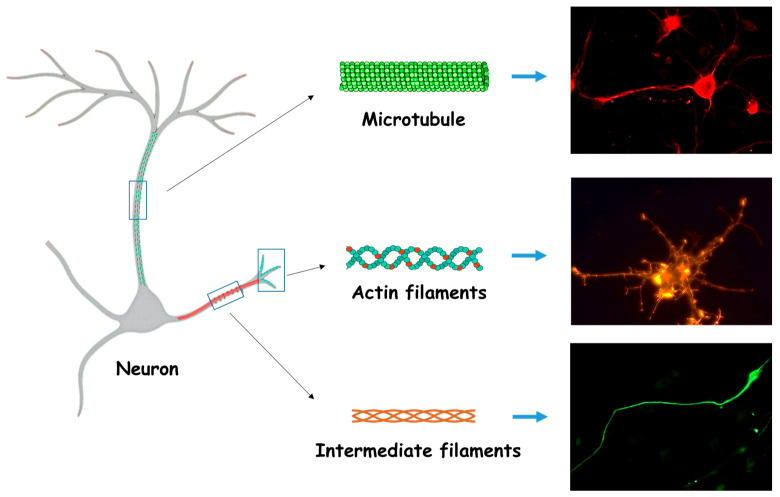
Neuronal cytoskeletal organization. Representative diagram showing the distribution of cytoskeletal components (microtubules, actin filaments, and intermediate filaments) within neuronal compartments. Microtubules are organized in polarized bundles that support axonal transport. Actin filaments are mainly concentrated in sites of morphological remodeling, such as dendritic spines and growth cones. Intermediate filaments (neurofilaments) are arranged along the axon, providing mechanical strength and maintaining axonal diameter. Each cytoskeletal element is labeled to illustrate its specific subcellular localization. Elements were adapted from Server Medical Art (https://smart.servier.com), licensed under Creative Commons Attribution 4.0 (https://creativecommons.org/licenses/by/4.0). The microscopy images shown on the right are original to the authors’ image bank and illustrate the normal distribution of the three principal components of the cytoskeleton. All images were acquired at a 60x magnification.

The cytoskeleton is a dynamic network of filaments structures that, in addition to determining cell shape, provides structural support, enables intracellular transport of molecules and organelles, and participates in motility, cell division, and adhesion, among others functions [72]. In neurons, this system acquires exceptional complexity due to their highly polarized morphology, characterized by long and thin processes such as axons and dendrites. The neuronal cytoskeleton is mainly composed of microtubules, actin filaments, and intermediate filaments (neurofilaments) (Figure 4). Together, these components form a highly organized network that not only maintains neuronal shape and structural stability, but also regulates essential dynamic processes, including axonal growth, synapse formation, and neuronal plasticity. Importantly, each cytoskeletal element exhibits specific cellular distribution patterns and function, as described below.

Microtubules are cylindrical polymers formed by α and β-tubulin heterodimers, whose assembly generates linear protofilaments. In immature neurons, microtubule nucleation is mediated by the microtubule-organizing center (MTOC), located at the centrosome. However, in mature neurons, microtubule assembly becomes non-centrosomal and occurs within axons or dendrites through alternative MTOCs, such as the Golgi apparatus or CAMSAPs complexes [73,74]. As a result, microtubules adopt a non-radial distribution. Microtubules are intrinsically polarized with minus (−) ends oriented toward the soma and plus (+) ends extending toward neurites [75], each exhibiting distinct dynamic properties. Although both ends can undergo growth and depolymerization, microtubule stability depends on guanosine-triphosphate hydrolysis (GTP) and is further modulated by microtubule-associated proteins (MAPs), such as Tau in axons and MAP2 in dendrites; microtubule (+)-end-binding proteins (+TIP) such as CLAPs and APC; and motor proteins including kinesins and dyneins. The expression and activity of MAPs vary according to the stage of neuronal development and subcellular location and are tightly regulated by post-translational modifications (e.g., phosphorylation, tyrosination, acetylation), which modulate their affinity for microtubule and, consequently, their dynamics [76]. In neurons, microtubules form the structural scaffold of axons and dendrites, enabling long-range transport of organelles and vesicles. Kinesins mediate anterograde transport toward the (+) end, whereas dyneins mediate retrograde transport toward the (−) end. Through these mechanisms, a wide variety of cellular components (including membrane fragments, intermediate filaments precursors, β-actin-encoding mRNA, neurotransmitters, and other essential elements) are efficiently mobilized [77,78].

Interestingly, members of the Rho family of small GTPases, belonging to the Ras superfamily, play a pivotal role in regulating MAPs and overall microtubules dynamic. These proteins act as molecular switches that alternate between an active GTP-bound state and an inactive GDP-bound state, enabling precise temporal and spatial control of cytoskeletal organization. Among the most extensively studied Rho-GTPases are RhoA, Cdc42 and Rac-1, which are essential regulators of cytoskeletal remodeling. Through downstream effectors such as mDia, EB1, APC, among others, Rho-GTPases coordinate the alignment and functional integration of microtubules and actin-filaments. This interplay ensures the proper execution of key cellular processes, including cell migration, polarity establishment, intracellular trafficking, and morphogenesis [79]. Given the high degree of interdependence between microtubules and actin filaments, these structures cannot be regarded as independent systems but rather as tightly co-regulated components of a unified and dynamic cytoskeletal network [80,81].

The actin cytoskeleton is composed of globular actin monomers (G-actin) that polymerize to form filamentous actin (F-actin) arranged in an asymmetric helical structure. Unlike microtubules or intermediate filaments, actin filaments are highly dynamic and semi-flexible, undergoing continuous cycles of assembly and disassembly through the addition or removal of G-actin monomers. This process is adenosine triphosphate (ATP)-dependent and tightly regulated by intracellular signaling cascades, allowing rapid cytoskeletal remodeling in response to cellular demands and environmental stimuli [82]. Because G-actin is polarized, F-actin also exhibits polarity, with a more dynamic (+) end and a relatively stable (−) end. F-actin forms a network that modulates and organizes the cytoarchitecture required for diverse cellular functions, including motility, endocytosis, absorption, and cell division [72].

In immature neurons, actin is predominantly distributed beneath plasma membrane and plays a crucial role in axonal initiation, guidance, and dendritic branching. Cycles of actin polymerization and depolymerization drive growth cone advancement and branch formation. In mature neurons, actin remains concentrated near the membrane but is also present in multiple compartments, including the soma, dendrites and their spines, axons, and synaptic terminals, where it contributes to maintaining neuronal polarity, and regulating neurites caliber [83]. In axons, recent studies have revealed the presence of a specialized actin network composed of patch-like structures and periodic actin-spectrin assemblies that support axonal structural integrity branching, and intracellular transport. In dendrites and dendritic spines, actin is highly abundant and plays a key role in shaping spine morphology and head-neck remodeling, processes essential for long-term potentiation (LTP) and long-term depression (LTD) [84]. Actin polymerization, Arp2/3-mediated branching, and cofilin-dependent turnover not only control spines remodeling but also promote synaptic consolidation [85]. In presynaptic terminals, actin participates in regulated movement of neurotransmitters-containing vesicles, whereas in postsynaptic compartments it modulates the dynamics and trafficking of synaptic receptors. In both cases, actin contributes to synaptic plasticity and the stabilization of intercellular connections [86].

Finally, intermediate filaments are composed of fibrous proteins that assemble into flexible and resilient structures, providing mechanical support and structural stability to cells. They confer tensile strength and preserve cellular integrity against mechanical stress. Unlike microtubules and actin filaments, intermediate filaments do not directly participate in cell motility. Their assembly is a highly ordered yet distinct from other cytoskeletal systems, as it does not require ATP or GTP and relies on lateral and longitudinal interactions between fibrous subunits. The basic monomer consists of a central α-helical rod domain flanked by non-helical head and tail regions that vary between cell types and regulate filament interactions and dynamics. Two monomers coil around one another in a parallel (head to head, tail to tail) fashion through their α-helical domains, forming a stable dimer, the fundamental assembly unit. Two dimers then associated in an antiparallel orientation, generating a neutral and nonpolar tetramer. Eight tetramers align laterally to form a cylindrical unit-filament, and the end-to-end addition of such units results in the formation of mature intermediate filaments [72]. Based on structure and sequence homology, intermediate filament proteins are classified into five major classes: classes I–IV are cytoplasmic, whereas class V comprises nuclear lamins (lamins A/C, B1 and B2). Types I and II include acidic and basic keratins that form heteropolymers. Type III includes homopolymers of peripherin, vimentin, desmin, and glial fibrillary acidic protein. Type IV comprises neurofilament heteropolymers (NFL, NFM and NFH), as well as internexin, synemin and nestin, which are expressed in the nervous system. Cytoplasmic intermediate filaments form a dense network that is predominantly perinuclear but extends towards the plasma membrane, contributing to mechanical integrity, intracellular organization, and cellular resilience [87].

In neurons, neurofilaments are predominantly distributed along the axonal axis, forming a longitudinal network that contributes to the regulation of axonal caliber (thickness) [88,89]. Some subtypes are also expressed in the soma and dendrites, although at lower levels than in axons. In immature neurons, additional proteins such as α-internexin or peripherin are present and can co-assemble with neurofilaments [90]. Recent studies have shown that neurofilaments act as compression and deformation shock absorbers within the axon, endowing it with viscoelastic properties that protect against mechanical stress. Beyond their structural role, neurofilaments also participate in slow axonal transport and in the positioning of organelles including mitochondria and endoplasmic reticulum. In several neurological disorders, neurofilaments subunits can be released into biological fluids following neuronal injury, making them valuable biomarkers of neurodegeneration [91,92].

It is well established that the cytoskeleton plays a critical role in aging and pathogenesis of neurodegenerative diseases, including AD [93,94], underscoring the importance of investigating its behavior under pathological conditions. In this context, cytoskeletal alterations induced by the presence of AβOs in neural tissue have been proposed as early events leading to cognitive impairment in AD. These alterations progressively disrupt neural connectivity, resulting in neurite fragmentation and synaptic loss. However, the precise pathological association between AβOs accumulation and cytoskeletal alterations remains incompletely understood.

## 6. AβOs and Their Effects on the Neuronal Cytoskeleton

Although the impact of AβOs on cytoskeletal structure and dynamics during the onset or early progression of AD remains incompletely understood, several experimental models have begun to clarify their effects on neuronal organization and function. Cytoskeletal alterations are now recognized as some of the earliest cellular events in AD pathogenesis [10,95]. Evidence indicates that during initial disease stages, AβOs interact with cytoskeletal components, inducing progressive structural and functional abnormalities that impair axonal transport and synaptic architecture (essential processes for efficient neural communication and critically implicated in AD development) [93,96]. Therefore, understanding the interactions between AβOs and cytoskeletal structures provides valuable insight into early pathological mechanisms and may contributing to the development of therapeutic strategies aimed at preserving cytoskeletal integrity and neuronal function in AD.

### 6.1. AβOs Effects on Microtubule Stability and Axonal Transport

During AD progression, the dynamics and stability of microtubules are altered both directly and indirectly by the presence of AβOs, as demonstrated by multiple studies [97,98]. Given that AβOs can be localized in both the intracellular and extracellular compartments, they are capable of exerting distinct toxic effects from each of these environments. As mentioned above, intracellular AβOs, being generated within the cell as a consequence of aberrant processing of APP in membranous organelles, can directly impair cytoskeletal organization and promote synaptic dysfunction. Evidence indicates that, during early stages, intracellular AβOs may predominate, whereas at more advanced stages extracellular AβOs become more abundant; however, a major limitation for performing comparative analyses in the lack of a defined quantitative ration between these two fractions, as their distribution varies depending on several factors, including the stage of the disease, the type of experimental model, and the detection techniques employed.

Microtubules are essential for neuronal morphology and synaptic plasticity. As previously noted, their dynamics depend on the association of MAPs, particularly Tau, which binds to and stabilizes microtubules. However, in AD, Tau becomes hyperphosphorylated, compromising its physiological function. Silva and colleagues [99] demonstrated that AβOs reduce the affinity of Tau for tubulin, promoting its abnormal phosphorylation and dissociation from microtubules, ultimately leading to microtubule instability. This destabilization was also associated with decreased ATP levels resulting from early mitochondrial alterations induced by Aβ internalization, findings that have been corroborated in pyramidal neurons from AD patients’ brains [100]. Notably, the same research group showed that microtubule disassembly further increases Tau phosphorylation, generating a self-perpetuating cycle of cytoskeletal disruption, later confirmed by other authors [101]. Consistent with this, AβOs disrupt axonal trafficking, affecting the transport of mitochondria and vesicles. These alterations occur early in the disease and contribute to functional neuronal decline. Microtubule destabilization has also been correlated with reduced kinesin-1 light chain (KLC1), impairing anterograde axonal transport, a process essential for neuronal homeostasis [102].

On the other hand, several Tau-independent mechanisms also contribute to microtubule destabilization. In this regard, Mota and colleagues [103] demonstrated that Aβ induces microtubule disassembly and neurite retraction through a NMDAR-dependent process, particularly via the GluN2B subunit. These authors reported that Aβ-induced transmembrane signaling can directly influence microtubules, affecting their stability and function [104], and correlating with structural neuronal deterioration. Importantly, these effects were prevented by NMDAR antagonists, confirming the pathological contribution of Aβ to cytoskeletal dysregulation through this pathway. Consistently, NMDAR-dependent mechanisms mediated by GSK-3β activation have been described in hippocampal neurons. Decker and coworkers [105] showed that either GSK-3β inhibition or NMDAR blockade restores axonal transport, reinforcing the relevance of this signaling pathway in AβOs-induced toxicity.

Recently, Kumar and colleagues [106] developed the microtubule-targeting radioligand [11C]MPC-6827 for PET imaging. In addition to efficiently crossing the blood–brain barrier, this radioligand displays high affinity for destabilized microtubules, as demonstrated in rodent (APP/PS1, P301S) and non-human primate models [107,108]. Its application in these experimental AD models provided information on the early effects of both Aβ and Tau on microtubule instability [109,110]. Notably, radiotracer uptake was significantly increased during early stages, prior to Aβ plaque formation and their detection by PET. These results suggest that AβOs may induce early microtubule dysregulation even in the absence of Tau pathology, as demonstrated in the 5×FAD mouse model, which lacks human Tau transgene expression.

In vivo studies have also demonstrated that microtubule disruption is an early and amplifying event in AD pathology. Sadleir and colleagues [111] reported that dystrophic neurites surrounding amyloid plaques exhibit marked microtubule loss, BACE 1 accumulation, and a local increase in Aβ generation, thereby promoting a pathological feedback loop.

Qu and colleagues hypothesized that Aβ not only activates signaling pathways that alter microtubule function but also promotes post-translational modifications (PTMs) in microtubule components [112]. Tubulin, in particular, undergoes several PTMs (including tyrosination-detyrosination, polyglutamylation, and acetylation) that modulate its functionality and stability [113], potentially triggering cellular stress. Previous studies have shown that detyrosinated tubulin can induce significant alterations in microtubule organization. In neuronal cells exposed to Aβ, sustained tubulin detyrosination has been observed [114], resulting in altered cell communication. Peris and coworkers [115] demonstrated that reduced activity of tubulin-tyrosine ligase (TTL), which is responsible for the re-tyrosination of α-tubulin, decreases microtubule dynamics, rendering them more stable. Consequently, this condition leads to dendritic spine alterations, synaptic dysfunction, and memory deficits. In human AD brains, a reduction in TTL activity has been reported, accompanied by the accumulation of detyrosinated tubulin and Δ2-tubulin, which correlated with disease severity and elevated levels of hyperphosphorylated Tau. Similar alterations have also been observed in neurons exposed to AβOs. Collectively, these findings suggest that AβOs toxicity, through multiple cellular mechanisms, contributes to early microtubule modifications that ultimately disrupt neuronal function in AD.

Considering that microtubule disruption induced by AβOs is an early event in AD, the use of microtubule-stabilizing agents represents an attractive strategy to address these abnormalities during the initial stages of the disease. In line with this concept, Penazzi and collaborators [116], using organotypic cultures derived from APP_SDL_ transgenic mice expressing human APP695 with three familial AD mutations, demonstrated that AβOs-induced disruption of microtubule dynamics leads to early dendritic spine loss. However, when the microtubule-stabilizing agent epothilone D (EpoD) was applied at a subnanomolar concentration (0.2 nM), sufficient to mildly stabilize microtubules, both dendritic spine loss and the associated morphological alterations were clearly reversed. Consistent with these observations, Fernandez-Valenzuela and collaborators [117] showed in the APP/PS1 transgenic mice model that early peripheral administration of EpoD (2 mg/kg) resulted in increased levels of acetylated α-tubulin, indicative of stable and polymerized microtubules. This effect was interpreted as an improvement in axonal transport and a reduction in pathological features, in contrast to untreated transgenic mice, which exhibited low levels of acetylated α-tubulin and depolymerized microtubules, as is typically observed in AD. Interestingly, EpoD treatment also reduced extracellular and intracellular Aβ accumulation in the hippocampus of these mice. One possible explanation for this effect is that the maintenance of efficient axonal transport, promoted by microtubule stabilization, facilitates lysosomal degradation of Aβ, thereby decreasing the abundance of its oligomeric forms. Taken together, these findings suggest that microtubule stabilization using compounds such as EpoD (despite certain limitations related to dosage, timing of administration, and treatment duration) could be considered a potential strategy to counteract early AβOs-induced cytoskeletal deterioration in AD.

### 6.2. AβOs Effects on Actin Microfilament Stability

Actin is considered the most dynamic of the three cytoskeletal components, a property that confers both physiological and pathological relevance. Alterations in actin dynamics are recognized as key events in several neurodegenerative diseases, including AD. Gao and colleagues [8] reported a biphasic effect of AβOs in SH-SY5Y cells. At moderate concentrations (750 nM to 1 µM) and exposure times up to 72 h, an increase in cell stiffness was detected by AFM, associated with actin polymerization, increased membrane tension, and Ca^2+^-dependent osmotic changes. In contrast, at higher concentrations (2–5 µM), AβOs induced pathological Tau phosphorylation, contributing to microtubule disassembly, and consequent loss of cell morphology. These findings provide biomechanical evidence of neurodegenerative changes induced by different AβOs concentrations and exposure durations, reflected by cytoskeletal alterations quantified through changes in Young’s modulus.

Among the mechanisms linking pathological actin remodeling with AβOs and AD, the LIMK1-cofilin-actin pathway is particularly relevant [118]. Under physiological conditions, LIM domain kinase 1 (LIMK1) phosphorylates cofilin at serine 3 (Ser3), inhibiting its depolymerizing activity, stabilizing actin filaments, and promoting spine enlargement [119]. This phosphorylation is reversed by F-actin-associated phosphatase Slingshot-1 (SSH1), which reactivates cofilin and restores its ability to bind actin filaments, a key mechanism for cytoskeletal dynamics [120]. Deficient LIMK1 activity has been associated with aging, impacting cytoskeletal regulation and cellular physiology. In the APP/PS1 mouse model, Woo and colleagues [121] demonstrated that AβOs promote cofilin dephosphorylation, leading to synaptic spines loss, impaired LTP, and memory deficits. Similarly, Kim and collaborators [122] reported cofilin dephosphorylation in cortical neuronal cultures exposed to AβOs. The same group found increased levels of dephosphorylated cofilin in AD patients compared with healthy controls, suggesting enhanced actin depolymerization due to elevated cofilin activity. Beyond phosphorylation status, excessive cofilin-actin complex formation can also trigger abnormal filament assembly and stabilization. Accordingly, cofilin-actin aggregates have been observed within senile plaques in both murine models and human AD brain tissue [123,124,125]. Interestingly, low (subnanomolar) concentrations of AβOs were shown to promote their formation [126]. Moreover, MAPs such as Tau have been detected alongside cofilin-actin aggregates, providing further evidence of cytoskeletal damage [127]. In the transgenic hAPP mouse model, reduced cofilin or Tau expression preserved synaptic function and memory plasticity [96,128].

It is important to note that cofilin activity on F-actin is concentration-dependent: at low concentrations it promotes filament destabilization, whereas at higher concentrations it can exert the opposite effect. Regardless of these conditions, cofilin has been proposed to play a significant role in several neurological diseases, including AD [129,130]. LIMK1 is activated by phosphorylation at Thr508 by members of the Rho GTPases family (including Cdc42, Rac1 or RhoA) [131]. This phosphorylation prevents cofilin binding to actin filaments, thereby inhibiting filament disassembly [132]. However, other reports indicate that ROCK and phosphorylated cofilin accumulate at high amounts in the brains of AD patients and murine models [133,134], suggesting that cofilin phosphorylation may also contribute to disease progression. Lee and colleagues [135] demonstrated that AβOs alter RhoA GTPase regulation through activation of the PyK2 tyrosine kinase (PTK2B), which phosphorylates Graf1c, inhibiting its regulatory function and maintaining sustained RhoA activation. This effect culminates in abnormal actin cytoskeleton reorganization, highlighting the PyK2-Graf1c-RhoA pathway as a mechanism involved in AβOs toxicity. In addition to RhoA, AβOs can also activate Rac1 and Cdc42, promoting PAK1 and LIMK1 phosphorylation and directly altering cofilin phosphorylation status [136]. Taken together, these data position AβOs as a central driver of AD pathology by promoting instability of the actin cytoskeleton.

Considering the above information, the LIMK1-cofilin-actin axis emerges as a promising candidate for the treatment of early synaptotoxicity induced by AβOs in AD. In this context, a therapeutic strategy aimed at modulating this pathway involves the use of ROCK inhibitors. According to the data reported by Sellers and collaborators [137], AβOs induce alterations in actin dynamics through activation of the Dkk1/Wnt-PCP →RhoA→ROCK signaling cascade. Pretreatment with the ROCK inhibitor Y-27632 in primary rat neuronal cultures exposed to AβOs effectively prevented dendritic spine loss. Similarly, the same study demonstrated that pretreatment with fasudil, a clinically approved ROCK inhibitor, blocked dendritic spines retraction in AβOs-treated cultures. Collectively, these findings indicate that ROCK inhibition can attenuate AβOs-associated synaptic structural deficits by antagonizing an actin-regulatory signaling axis activated during the early stages of AD.

In parallel, Henderson and coworkers [138], seeking to identify more specific molecular targets underlying AβOs-induced dendritic spine degeneration, reported that specifically the ROCK2-LIMK1 pathway plays a critical role in maintaining spine integrity. Using rat hippocampal neuron cultures exposed to AβOs, they showed that pharmacological inhibition of LIMK1 with SR7826 conferred dendritic spine resilience, preventing the rapid spine degeneration observed under AβOs exposure alone. These findings suggest that LIMK1 inhibition may preserve dendritic spine integrity under conditions of AβOs-induced actin stress and support its potential as an early intervention strategy for preserving synapses, warranting further validation in in vivo models.

### 6.3. AβOs Effects on Neurofilament Stability and Early Axonal Damage

Neurofilament integrity depends on dynamic interactions with microtubules through associated proteins, including MAPs and kinases that regulate axonal transport. Although neurofilaments have been less investigated than other cytoskeletal elements in AD, growing evidence supports their involvement in disease pathogenesis. Several studies indicate that neurofilament light chain (NFL), which plays an important role in axonal and dendritic growth [139,140], can also serve as a biomarker of neurodegeneration [141,142]. Elevated NFL levels have been reported in both cerebrospinal fluid (CSF) and plasma in neurodegenerative disorders, including AD, as well as in animal models [142,143,144], correlating with axonal damage, neuronal injury, and disease severity [145]. Accordingly, Kang and colleagues [146] demonstrated in humans and in a transgenic APP model (McGill-Thy-APP Tg rats) that Aβ42/40 ratio induces an increase in NFL levels, accompanied by reduced gray matter density in vulnerable brain regions (hippocampus and neocortex) in AD. Importantly, the Tg-rat model does not express Tau protein, allowing exclusion of Tau-related effects. NFL concentrations were increased in CSF from Tg-rats compared with WT-rats, and when aligned with the timing of Aβ plaque appearance, the early rise in NFL was associated with AβOs. These findings support the notion that AβOs alone are sufficient to induce neuronal injury (particularly at the cytoskeletal level) with minimal cell death, consistent with early disease stages. Notably, although elevated NFL reflects neuronal injury rather than exclusively AβOs-induced toxicity, they can be used as an early marker of damage in both aging and multiple pathologies included AD. Combined with complementary tools such as neuroimaging studies, NFL quantification could offer a valuable strategy for early AD detection.

Finally, reactive oxygen species (ROS) are abundant in AD and can affect cellular function through multiple mechanisms. Importantly, ROS are now recognized not only as damaging molecules but also as modulators of diverse signaling and regulatory pathways [147]. In this context, Gardiner and collaborators [148] reported that neurofilaments become abnormally phosphorylated under oxidative stress, leading to their aggregation. Together with AβOs and pathological aggregates of hyperphosphorylated Tau, these alterations disrupt intraneuronal transport and impair cell–cell communication in AD as well as other neurological diseases.

In contrast to pharmacological approaches aimed at preventing or reducing AβOs-induced alterations in other cytoskeletal components, there are currently no approved drugs capable of restoring or maintaining damaged neurofilaments. Nevertheless, clinical studies have demonstrated that interventions targeting Aβ reduction, particularly through the use of anti-Aβ monoclonal antibodies such as lecanemab and donanemab (currently the main pharmacological class exerting an indirect impact on neurofilaments) have shown beneficial effects. In addition to reducing cerebral amyloid burden, attenuating neuronal damage, and slowing cognitive decline, these therapies have also been reported to decrease plasma NFL levels when administered during the early stages of the disease [149,150]. Collectively, these findings suggest that NFL is better suited as a pharmacodynamic biomarker to facilitate the monitoring of therapeutic efficacy, rather than as a direct therapeutic target in AD.

## 7. Conclusions

**Figure 5:**
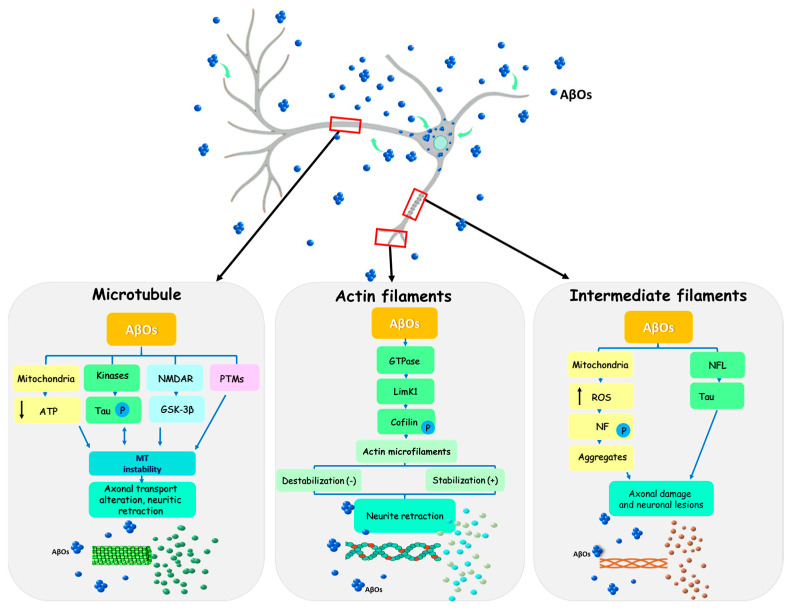
Schematic representation of neuronal cytoskeleton alterations induced by AβOs. The illustration depicts a neuron (gray) with preserved morphology, surrounded by and containing endogenous AβOs (blue) within an AD brain. The curved arrows (green) indicate that AβOs can be internalized into the cell. Exposure to AβOs promotes remodeling of the cytoskeletal network, resulting in changes in its organization and dynamics. Although these early alterations do not immediately lead to neuronal death, they contribute to functional impairment, primarily through the disruption of intercellular communication driven by abnormal dendritic retraction a typical hallmark of AD.

The neuronal cytoskeleton is an ordered network composed of microtubules, actin filaments and intermediate filaments that are intrinsically interconnected and play essential roles in cellular organization, intracellular transport, and neuronal homeostasis. However, disruption of this system is closely associated with the development of neurodegenerative diseases. In this review, we summarize some of the most relevant alterations affecting the neuronal cytoskeleton during the initial stages of AD (Figure 5). Accumulating evidence indicates that AβOs profoundly and early disrupt the development and organization of this structural system, triggering a cascade of dysfunctions that affect multiple organelles, including the Golgi apparatus, endoplasmic reticulum and mitochondria, ultimately leading to functional impairment and progressive neuronal degeneration. Accordingly, several research groups propose that cytoskeletal alterations represent one of the most relevant early events in the pathophysiology of AD [151,152].

Consequently, the detailed study of cytoskeleton dynamics under both physiological conditions and AβOs-induced stress is emerging as a critical axis for understanding early disease mechanisms. Likewise, the identification of molecular targets capable of selectively and safely modulating cytoskeletal stability could open new therapeutic opportunities. However, direct manipulation of the cytoskeleton presents significant challenges due to its fundamental involvement in multiple essential processes for neuronal viability. Therefore, it is imperative to expand research efforts using advanced imaging technologies, high-resolution molecular tools, and experimental models that allow a more precise elucidation of the temporal sequence and functional relevance of these alterations. A deeper understanding of the interactions between AβOs, the cytoskeleton, and early AD progression will not only strengthen our knowledge of disease etiopathogenesis but will also facilitate the development of diagnostic and therapeutic strategies targeting early stages of the disease, when intervention may be most effective.

## 8. Future Directions

Based on the evidence reviewed and our research experience in this field, future perspectives should focus on the integration of multidisciplinary approaches aimed at elucidating the molecular mechanisms linking AβOs to alterations in cytoskeletal structure and dynamics that drive neuronal damage during the early (presymptomatic) stages of AD.

The continued development and application of super-resolution imaging technologies, conformational biosensors, and multi-omics approaches remain essential to facilitate the identification of early molecular signatures associated with modifications in tubulin, cofilin, and neurofilaments, as well as with disruptions in axonal transport and synaptic plasticity. In addition, the validation of biomarkers such as NFL, in combination with markers of microtubule instability or actin remodeling, could improve early detection and monitoring of disease progression. Finally, the development of novel therapeutic strategies targeting key regulatory nodes (such as the LIMK1–cofolin–actin pathway and the modulation of TTL) together with cellular and animal models and early-phase clinical trials, will enable a more accurate evaluation of the potential of targeting cytoskeletal integrity as a preventive or disease-modifying approach in AD.

## Data Availability

No new data were created or analysed in this study. Figure 2 illustrate advanced pathological Aβ lesions in the AD. Figure 4 illustrate the normal distribution of the three principal components of the cytoskeleton. Data sharing is not applicable to this article.
